# Hepatic–Renal Biomarker Clustering Identifies Subclinical Acclimatization Phenotypes at High Altitude

**DOI:** 10.3390/life16101595

**Published:** 2026-09-24

**Authors:** Yonghao Ou, Xiaoyu Li, Yongjun Jing, Hao Li

**Affiliations:** 1 Xizang Autonomous Region Key Laboratory for High Altitude Brain Science and Environmental Acclimatization, Xizang University, Lhasa 850000, China; 2 Plateau Brain Science Research Center, Xizang University, Lhasa 850000, China; 3 China Railway No. 10 Engineering Group Co., Ltd., Shuntai Plaza, Jinan High-Tech Zone, Jinan 250101, China

**Keywords:** high altitude, altitude acclimatization/adaptation index, hepatic–renal biomarkers, hierarchical clustering

## Abstract

Accurate assessment of high-altitude acclimatization is essential for identifying individuals at risk of maladaptation, yet current strategies relying on single-organ biomarkers or subjective symptom scores fail to capture the multi-organ physiological response to hypoxia. This study aimed to identify distinct acclimatization phenotypes through unsupervised hierarchical clustering of five routinely available hepatic and renal biomarkers and to evaluate their association with an objective index of high-altitude acclimatization. A total of 184 healthy adults residing in Lhasa (3650 m) were recruited. Serum creatinine (CR), uric acid (UA), alanine aminotransferase (ALT), aspartate aminotransferase (AST), and total bilirubin (TBIL) were standardized and submitted to hierarchical clustering. The altitude acclimatization/adaptation index (AAI), defined as the ratio of peripheral oxygen saturation (SpO_2_) to hematocrit (HCT), was used as an criterion of acclimatization status. Cluster stability was assessed through multiple sensitivity analyses, and a general linear model evaluated the association between cluster assignment and AAI. Two clusters were identified: a Relatively Healthy cluster (*n* = 152) and a Metabolically Abnormal cluster (*n* = 32), differing significantly across all five indicators and in AAI. Sensitivity analyses supported the robustness of this two-cluster structure. General linear model results showed that, after adjustment for sex, race, age, and body mass index, cluster assignment remained significantly associated with AAI (*p* = 0.029), with adjusted means of 1.97 versus 1.84. Both values exceeded the maladaptation threshold of 1.72, underscoring that the panel detects meaningful acclimatization differences within the conventionally well-acclimatized range. Five hepatic and renal biomarkers stratify high-altitude residents into distinct acclimatization phenotypes by capturing subclinical metabolic strain within the conventionally well-acclimatized range and identify actionable intervention targets for advancing precision management of high-altitude acclimatization.

## 1. Introduction

With the improvement of infrastructure and the enhancement of public service levels in high-altitude areas, an increasing number of people are moving to or temporarily staying in these regions for diverse purposes such as study, work, border defense, scientific research, and tourism. Unsurprisingly, the hypoxic environment at high altitude imposes substantial physiological strain, primarily hypoxic injury to visceral organs [1,2,3]. Among these visceral organs, the liver and kidneys are particularly susceptible to hypoxic injury, given their high metabolic demands and rich blood perfusion [4,5,6,7]. Therefore, monitoring changes in both hepatic and renal function indicators is of great value for assessing the progress of high-altitude acclimatization and detecting early maladaptive responses.

Building on this recognition, a considerable body of research has utilized hepatic and renal biomarkers to assess the status of high-altitude acclimatization [8,9,10,11]. However, three key limitations persist in the current literature. First, existing studies tend to focus on single biomarkers from either the liver or the kidney in isolation, which fails to capture the multidimensional nature of physiological adaptation to hypoxia [10,12,13]. Second, hepatic and renal indicators reflect distinct physiological dimensions of hypoxic stress. The liver primarily mirrors metabolic and detoxification processes, whereas the kidney responds to alterations in perfusion, fluid–electrolyte balance, and excretion, yet few attempts have been made to integrate these organ-specific signals into a unified analytical framework [8,14,15,16,17]. Third, it remains largely unknown which specific indicators, or which combinations of indicators, can most effectively discriminate between individuals with favorable acclimatization and those exhibiting maladaptive responses [8,18]. To address these limitations, the present study employs cluster analysis to simultaneously incorporate multiple hepatic and renal parameters, with the goal of identifying distinct acclimatization phenotypes and determining sensitive biomarker profiles for high-altitude adaptation.

A critical prerequisite for linking hepatic and renal biomarker profiles to high-altitude acclimatization is an objective criterion for classifying individuals as well-acclimatized or maladapted, given that previous studies largely relied on subjective symptom-based questionnaires [19,20,21,22]. A recently proposed index, the altitude acclimatization/adaptation index (AAI), defined as the ratio of pulse oxygen saturation to hematocrit (SpO_2_/HCT), provides such a tool [23]. Physiologically, it captures the efficiency of oxygen delivery relative to the hemoconcentration burden imposed by chronic hypoxia. The AAI has been validated to discriminate effectively between well-acclimatized and poorly acclimatized individuals, with lower values indicating maladaptation. Compared with questionnaires, the AAI is continuous, observer-independent, and biologically interpretable, and was therefore adopted in the present study as the criterion for defining acclimatization status.

In summary, the present study integrated unsupervised cluster analysis of five hepatic and renal biomarkers with the objective AAI criterion to identify distinct acclimatization phenotypes and their biochemical signatures. This integrative strategy offers a pathway from symptom-based assessment toward biomarker-driven identification of intervention targets for high-altitude acclimatization in populations ascending to or residing in hypoxic environments.

## 2. Materials and Methods

### 2.1. Participants

A total of 184 healthy participants (mean age: 29.72 ± 8.44 years) residing in Lhasa were recruited for this study between 1 September and 30 September 2024. Participants were eligible for inclusion if they (1) had no history of cardiovascular, cerebrovascular, respiratory, or mental disorders; (2) had normal vision or corrected-to-normal vision, with no color vision deficiency; and (3) were not taking any regular medications at the time of the study. Migrants were defined as those born and raised below 1500 m, and natives as those at or above 1500 m. The participants consisted of 43 male and 141 female subjects, including 94 migrants and 90 natives. The study protocol was reviewed and approved by the Ethics Committee of Xizang University (Approval Number: ZDYXLL2024003), and the procedures were conducted in strict accordance with the Declaration of Helsinki. All participants were fully informed of the experimental procedures and potential risks, and written informed consent was obtained prior to their participation.

### 2.2. Experimental Procedure

All testing procedures were conducted on a single day under standardized conditions. Upon arrival at the hospital at 7:30 AM, participants were thoroughly briefed on the study protocols, and written informed consent was obtained. Following a 10 h overnight fast (with water permitted), baseline venous blood samples were collected. Participants then consumed a standardized breakfast and rested for 30 min. During this rest period, demographic, anthropometric, and baseline physiological data were recorded, including height, weight, and oxygen saturation (SpO_2_). Height and weight were measured to the nearest 0.01 cm and 0.01 kg, respectively, and body mass index (BMI) was calculated. SpO_2_ was measured using a finger clip pulse oximeter (YX301, Yuwell, Danyang, Jiangsu, China) while the participants were in a state of rest.

### 2.3. Blood Sampling and Biochemical Analyses

A 5 mL fasting venous blood sample was drawn from the antecubital vein by a certified phlebotomist at Tibet Fukang Hospital (Lhasa, China). After clotting at room temperature for 2 h and centrifugation (1500× *g*, 10 min), serum was aliquoted and stored at −80 °C until analysis. Complete blood count, including hemoglobin (Hb) and HCT, was measured in whole blood using an automated hematology analyzer (BC-5390; Mindray, Shenzhen, China) with manufacturer-provided reagents within 4 h of collection and daily internal quality control. Serum biochemical parameters, such as creatinine (CR) and uric acid (UA), were measured by the clinical laboratory of the same hospital using standard automated biochemical assays with routine internal quality control and calibration. The AAI was calculated as SpO_2_/HCT [23].

### 2.4. Statistical Analysis

To ensure clinical applicability, we selected feature variables based on their established physiological relevance and accessibility in routine clinical practice. Five core biochemical indicators were selected: CR, UA, alanine aminotransferase (ALT), aspartate aminotransferase (AST), and total bilirubin (TBIL). These five indicators represent the core dimensions of renal (CR, UA) and hepatic (ALT, AST, TBIL) function [8,11,24]. Other available parameters such as urea, AST/ALT ratio, and bilirubin fractions were considered derivative or indirect reflections of these core pathways and were therefore not included to avoid redundancy and multicollinearity. All variables were standardized to z-scores before clustering. A Manhattan distance matrix was computed, and clustering was carried out using Ward’s minimum variance method [25,26]. The optimal number of clusters was determined by examining the silhouette coefficient for *k* = 2 to 8; *k* = 2 yielded the highest average silhouette width (0.5035) and was retained for its clinical interpretability. A line plot of silhouette coefficients across *k* = 2 to 8 is provided in Supplementary Figure S1. Cluster stability was assessed through several sensitivity analyses: (1) comparison with alternative clustering approaches (Euclidean distance with Ward’s minimum variance method or complete linkage) using the adjusted Rand index (ARI); (2) exclusion of participants with ALT > 80 U/L or UA > 540 μmol/L, based on established clinical thresholds for significant hepatocellular injury and severe hyperuricemia requiring intervention [27,28], followed by re-clustering; (3) validation with partitioning around medoids (PAM, k-medoids) using the same Manhattan distance; and (4) bootstrap resampling (50 iterations) to compute Jaccard similarity coefficients for each cluster, a Jaccard value > 0.75 was considered stable [29,30]; (5) sex-stratified clustering and sex-adjusted z-score clustering were additionally performed to evaluate the influence of sex on the cluster structure. In addition, 95% confidence intervals for the Jaccard coefficients were derived from the bootstrap distribution. Internal validation was performed using a 70/30 sample-split cross-validation, in which the training set was used to derive cluster centers and the test set was assigned to the nearest center based on Manhattan distance; agreement with the original clustering was quantified by ARI. The clustering results were visualized using principal component analysis (PCA) scatter plots with 95% confidence ellipses generated by the factoextra and ggsci packages.

After obtaining the cluster assignments, descriptive statistics were calculated for all 184 participants. Continuous variables were presented as mean ± standard deviation (SD), and categorical variables as frequencies and percentages. Baseline comparisons between the two clusters (Cluster 1 vs. Cluster 2) were performed using Welch’s two-sample *t*-test for continuous variables and Fisher’s exact test for categorical variables, given the imbalance in sample sizes and small expected cell counts. Non-normally distributed variables (Shapiro–Wilk *p* < 0.05) were additionally analyzed using Mann–Whitney *U* tests as sensitivity analyses. To account for multiple comparisons, all *p* values from the between-cluster comparisons were adjusted using the Benjamini–Hochberg false discovery rate (FDR) method. SpO_2_ and HCT, the two components of AAI, were also compared separately between clusters.

To evaluate the independent effect of the derived clusters on the AAI, an analysis of covariance (ANCOVA) model was fitted. The independent variable was the cluster assignment (Cluster 1 vs. Cluster 2) and the outcome variable was AAI. The following covariates were included a priori based on clinical relevance: sex (male/female), race (migrant/native), age (years), body mass index (BMI, kg/m^2^). To assess whether the cluster effect on AAI was modified by sex or race, interaction terms (cluster × sex and cluster × race) were initially included. As neither interaction term reached statistical significance, the final model excluded interaction terms, and all reported estimates are based on this model. Model assumptions were assessed graphically by inspecting residual Q-Q plots and residuals-vs-fitted plots (see Supplementary Figure S2). Type III ANOVA was used to test the effect of each predictor while adjusting for all other predictors. Least-squares (marginal) means and their 95% confidence intervals (CIs) were estimated for the two clusters using the emmeans package. Pairwise comparisons were performed with Tukey’s adjustment. A two-tailed *p*-value < 0.05 was considered statistically significant. Post hoc power analysis for the full ANCOVA model was performed using the observed Cohen’s *f*^2^ and a two-tailed α = 0.05. Data visualization was performed using ggplot2 and ggdist to create raincloud plots. All analyses were performed using R version 4.5.3 (R Foundation for Statistical Computing, Vienna, Austria; R Core Team, 2025) with the cluster, fpc, mclust, car, and emmeans packages.

## 3. Results

### 3.1. Clustering Analysis

Hierarchical cluster analysis was performed using five biochemical indicators: CR, UA, ALT, AST, and TBIL. The analysis yielded two clusters: Cluster 1 (*n* = 152) and Cluster 2 (*n* = 32). Independent-samples *t*-tests confirmed that the two clusters differed significantly in all five clustering indicators (*p* < 0.001). Based on these characteristics, the clusters were designated as “Relatively Healthy” (Cluster 1) and “Metabolically Abnormal” (Cluster 2). The average silhouette width was 0.5035, indicating reasonable separation between clusters (see Figure 1).

The robustness of the clustering was evaluated through multiple sensitivity analyses. Comparison with Euclidean distance combined with Ward’s minimum variance method yielded an ARI of 0.36; given the right-skewed distributions and potential outliers, Manhattan distance was selected a priori as less sensitive to extreme values. Comparison with Euclidean distance + complete linkage showed very low agreement (ARI = 0.08), as that method produced a highly unbalanced solution with one cluster containing only 4 participants. In contrast, removing fifteen outliers (ALT > 80 U/L or UA > 540 μmol/L) yielded an ARI of 0.89, indicating almost perfect agreement with the original clustering. Agreement with partitioning around medoids (PAM) was moderate (ARI = 0.76), and the PAM silhouette coefficient was 0.4461. Bootstrap resampling (50 iterations) produced Jaccard similarity coefficients of 0.82 (95% CI: 0.70–0.83) for Cluster 1 and 0.62 (95% CI: 0.48–0.75) for Cluster 2, demonstrating high stability of the larger cluster and acceptable stability of the smaller cluster. In addition, sex-stratified and sex-adjusted clustering yielded an ARI of 0.016 relative to the primary solution, indicating that the cluster structure was largely sex-dependent. Finally, a 70/30 sample-split cross-validation yielded an ARI of 0.81, supporting the reproducibility of the cluster structure.

### 3.2. Descriptive Statistics

Descriptive statistics for the two clusters are summarized in Table 1. After FDR correction, the Metabolically Abnormal cluster (Cluster 2) showed significantly higher levels of urea, CR, UA, ALT, AST, TBIL, direct bilirubin (DBIL), indirect bilirubin (IBIL), albumin (ALB), and body mass index (BMI) compared with the Relatively Healthy cluster (Cluster 1) (all *p* < 0.05), whereas the AST/ALT ratio was significantly lower (*p* < 0.001). No significant differences were observed for total protein (TP), globulin (GLOB), or age (all *p* > 0.05). Regarding altitude acclimatization parameters, HCT was markedly higher in Cluster 2 (54.13% vs. 44.66%, *p* < 0.001), while SpO_2_ did not differ significantly after FDR correction (90.31% vs. 91.41%, *p* = 0.059). The AAI was significantly lower in Cluster 2 (1.83 vs. 1.96, *p* = 0.038). Regarding demographic characteristics, Cluster 2 consisted predominantly of males (93.8% vs. 8.6%, *p* < 0.001) and had a higher proportion of migrants (68.8% vs. 47.4%, *p* = 0.044).

### 3.3. General Linear Model (ANCOVA) for AAI

To evaluate the independent effect of cluster assignment on the AAI, a general linear model (ANCOVA) was fitted with sex, race, age, and BMI as covariates. The overall model was highly significant (*F*(5, 178) = 39.71, *p* < 0.001) and explained 52.7% of the variance in AAI (adjusted *R*^2^ = 0.514). The results demonstrated that cluster assignment was a significant predictor of AAI (*F*(1, 178) = 4.82, *p* = 0.029). The estimated marginal means were 1.97 (95% CI: 1.91–2.02) for Cluster 1 (Relatively Healthy) and 1.84 (95% CI: 1.76–1.92) for Cluster 2 (Metabolically Abnormal), corresponding to a mean difference of 0.13 (95% CI: 0.01–0.25; *p* = 0.029). Thus, individuals in Cluster 2 had significantly lower AAI, indicating poorer high-altitude acclimatization compared with Cluster 1. Neither the cluster × sex interaction (*p* = 0.450) nor the cluster × race interaction (*p* = 0.871) was significant, indicating that the cluster effect on AAI was consistent across sex and race. Sex was a strong independent predictor, with males showing lower AAI than females (*F*(1, 178) = 16.44, *p* < 0.001). Race had an even stronger effect, with native residents showing higher AAI than migrants (*F*(1, 178) = 56.90, *p* < 0.001). Age and BMI were not significant predictors (*p* > 0.05; see Figure 2). Post hoc power analysis for the full ANCOVA model indicated a power of >0.99 (Cohen’s *f*^2^ = 1.12, α = 0.05, two-tailed).

## 4. Discussion

The present study was designed to identify clinically meaningful acclimatization phenotypes by integrating unsupervised hierarchical clustering of five core hepatic and renal biomarkers with the objective AAI. First, the clustering results revealed two distinct clusters, a “Relatively Healthy” cluster (Cluster 1) and a “Metabolically Abnormal” cluster (Cluster 2), that differed markedly in all five clustering variables, namely CR, UA, ALT, AST, and TBIL, as well as in a range of other biochemical and demographic characteristics. Second, multiple sensitivity analyses, including re-clustering after removing individuals with clinically significant elevations in ALT or UA, alternative algorithm validation, and bootstrap resampling, consistently supported the robustness of this two-cluster structure. Finally, more importantly, general linear model results showed that cluster assignment remained a significant independent predictor of AAI after adjusting for sex, race, age, and BMI. This finding demonstrates that the two clusters are not merely statistical artifacts but capture distinct hepatic–renal response patterns that are differentially associated with high-altitude acclimatization status.

The distinct biochemical profiles of the two clusters can be understood through the coordinated physiological burden that high-altitude hypoxia imposes on the liver and kidney. In the Metabolically Abnormal cluster, the concurrent elevation of all five indicators suggests an integrated multi-organ stress response rather than isolated organ-specific dysfunction. Central to this response is the marked hyperuricemia, which exemplifies the dual hepatic–renal mechanism underlying the observed metabolic disturbance. Hypoxia upregulates xanthine oxidase and adenosine deaminase in the liver, accelerating purine catabolism and uric acid production, while simultaneously downregulating renal urate transporters OAT1 and OCT1, thereby impairing uric acid excretion [9,10]. This coupled hepatic overproduction and renal underexcretion makes uric acid a particularly sensitive sentinel of the hepatic–renal metabolic axis under hypoxic stress. The same oxidative stress that drives purine metabolism also underlies the hepatocyte membrane injury reflected in elevated ALT and AST, which experimental studies have shown to rise by approximately 30% and 25% respectively, accompanied by a 42% increase in hepatic malondialdehyde [8]. Consistent with this oxidative picture, the elevated creatinine and total bilirubin in Cluster 2 further indicate reduced glomerular filtration, attributable to hemoconcentration and decreased renal plasma flow [17], and altered hepatic bilirubin handling linked to impaired renal recovery after high-altitude exposure [31]. Collectively, these interconnected alterations support the concept of a hepatic–renal metabolic axis in high-altitude acclimatization, wherein hypoxia-driven oxidative stress simultaneously triggers hepatic enzyme induction, purine metabolism activation, and renal hemodynamic and tubular transport changes [6]. By simultaneously capturing these five signals, the clustering algorithm effectively separated individuals who maintain metabolic homeostasis under hypoxic stress from those already exhibiting early, subclinical evidence of hepatic–renal metabolic strain.

A particularly instructive finding of the present study is that the mean AAI values in both clusters (1.96 and 1.83) remained above the previously established cut-off of 1.7228 for maladaptation [23], yet the between-cluster difference remained significant after covariate adjustment. This observation points to a fundamental limitation of relying on any single index, even a composite one, to capture the full spectrum of acclimatization. Although the AAI integrates SpO_2_ and HCT and thereby overcomes the well-recognized confounds inherent in either measure alone, such as anthropometric variability, measurement inconsistency, and non-acclimatization-related fluctuations [32,33,34], it remains, at its core, an index of the oxygen delivery–erythrocytosis balance. A growing body of evidence, however, indicates that high-altitude acclimatization extends well beyond adjustments in oxygen delivery to encompass profound remodeling of tissue oxygen utilization and metabolic regulation [35,36]. At the cellular level, the HIF-mediated transcriptional response to hypoxia suppresses oxidative metabolism and promotes glycolytic capacity, metabolic shifts that can be initiated before any clinically detectable change in oxygen saturation or hematocrit [35,36]. Consistent with this temporal sequence, subclinical renal dysfunction and elevated oxidative stress markers have been detected within days of high-altitude exposure, well before conventional oxygenation indices reach pathological thresholds [34,37]. The hepatic and renal biomarkers that defined our clusters are directly linked to these early hypoxia-driven metabolic disturbances. Oxidative stress, a hallmark of high-altitude exposure, simultaneously impairs hepatocyte membrane integrity, raising serum transaminases, and disrupts purine metabolism, leading to hyperuricemia through both enhanced hepatic production and reduced renal excretion of uric acid [8,9,10]. This dual hepatic–renal burden exemplifies the integrated metabolic strain captured by our five-indicator panel. Consistent with this view, multi-marker approaches that combine complementary host-response pathways have been shown to outperform single-analyte assessments in predicting high-altitude illnesses [10,38]. Our clustering thus provides a biochemical readout of the metabolic cost of maintaining oxygen homeostasis, a dimension of acclimatization that is not reflected by the AAI alone. The ability to detect a significant AAI gradient between two groups that both fell within the conventionally defined well-acclimatized range, therefore, indicates that the panel identifies subclinical metabolic strain that precedes overt maladaptation, positioning hepatic–renal clustering as a sensitive complement to AAI-based screening.

The demographic composition of the two clusters, with Cluster 2 comprising predominantly males and a higher proportion of Han Chinese migrants, aligns with well-established sex- and ancestry-based differences in high-altitude adaptation. Males exhibit a substantially greater erythropoietic drive and uric acid burden, driven in part by testosterone-mediated stimulation of xanthine oxidase and higher erythrocyte turnover [39,40]. With respect to ancestry, Tibetans have acquired, through millennia of natural selection, functional variants in *EGLN1* and *EPAS1* that promote HIF degradation under hypoxia, thereby blunting erythropoiesis and protecting against excessive polycythemia [41]. Han migrants, who lack these protective alleles, show persistently elevated hematocrit, incomplete cardiac normalization, and a greater magnitude of phenotypic change relative to Tibetans [42,43], alongside evidence of accelerated epigenetic aging that underscores the biological cost of acclimatization in non-adapted populations [44]. Critically, however, the independent effect of cluster assignment on AAI persisted after adjustment for sex and race. This indicates that the hepatic–renal biochemical profile captures residual inter-individual variation in the metabolic cost of maintaining oxygen homeostasis that is not reducible to demographic categories. The clustering approach thus provides a biomarker-based stratification that complements, rather than recapitulates, the demographic risk factors long recognized in high-altitude medicine.

Several limitations should be acknowledged. First, the identified clusters were highly collinear with sex, with the Metabolically Abnormal cluster comprising almost exclusively males, indicating that the phenotypes largely reflect sex-specific biochemical profiles rather than sex-independent metabolic traits; validation in sex-balanced cohorts is therefore essential. Second, the cluster structure was sensitive to the choice of distance metric, and the smaller cluster showed only moderate stability, falling below the predefined threshold, which may reflect its limited sample size and internal heterogeneity. Third, detailed altitude exposure history and additional biomarkers such as erythropoietin were not collected, limiting full characterization of acclimatization trajectories. Fourth, the single-center design and modest sample size, particularly for the smaller cluster, may limit generalizability, and the internal cross-validation provides only partial support; external validation in independent, multi-ethnic cohorts across a range of altitudes is required. Finally, the cross-sectional design and absence of longitudinal clinical outcomes preclude causal inference and direct evaluation of predictive utility, warranting prospective studies with repeated measurements and clinical endpoints to establish the clinical value of this biomarker-based stratification strategy.

## 5. Conclusions

The present study demonstrates that unsupervised hierarchical clustering of five routinely available hepatic and renal biomarkers, namely CR, UA, ALT, AST, and TBIL, identifies two distinct acclimatization phenotypes that are associated with objectively measured high-altitude adaptation. Critically, the hepatic–renal biomarker panel detected significant between-cluster differences in acclimatization status even within the conventionally defined well-acclimatized range, indicating that it captures subclinical metabolic strain that precedes overt maladaptation and thus provides incremental stratification beyond single-index assessment. These findings identify actionable intervention targets for high-altitude acclimatization, advancing biomarker-driven precision management for populations ascending to or residing in hypoxic environments.

## Figures and Tables

**Figure 1:**
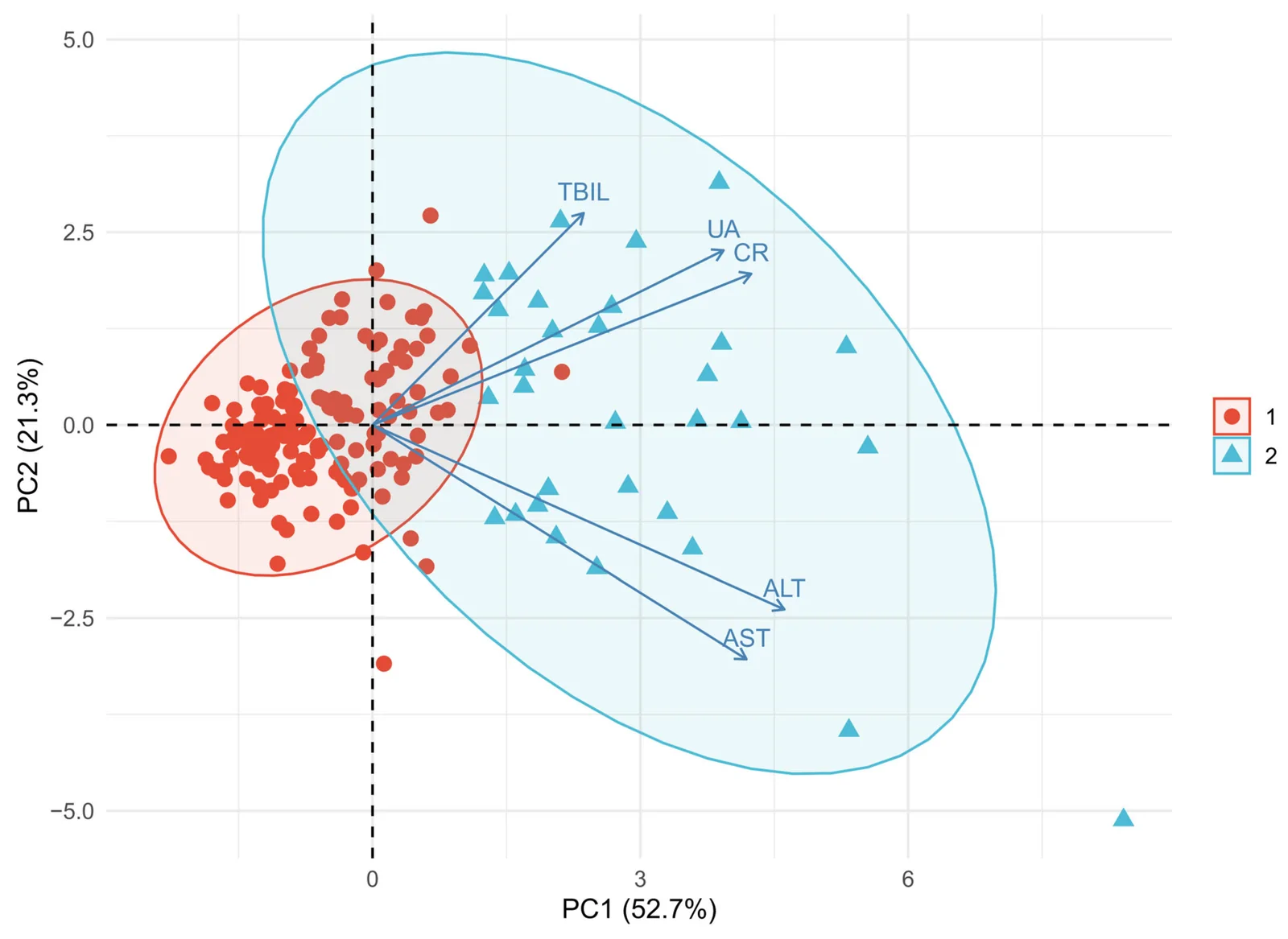
PCA scatter plot of the two clusters with 95% confidence ellipses.

**Figure 2:**
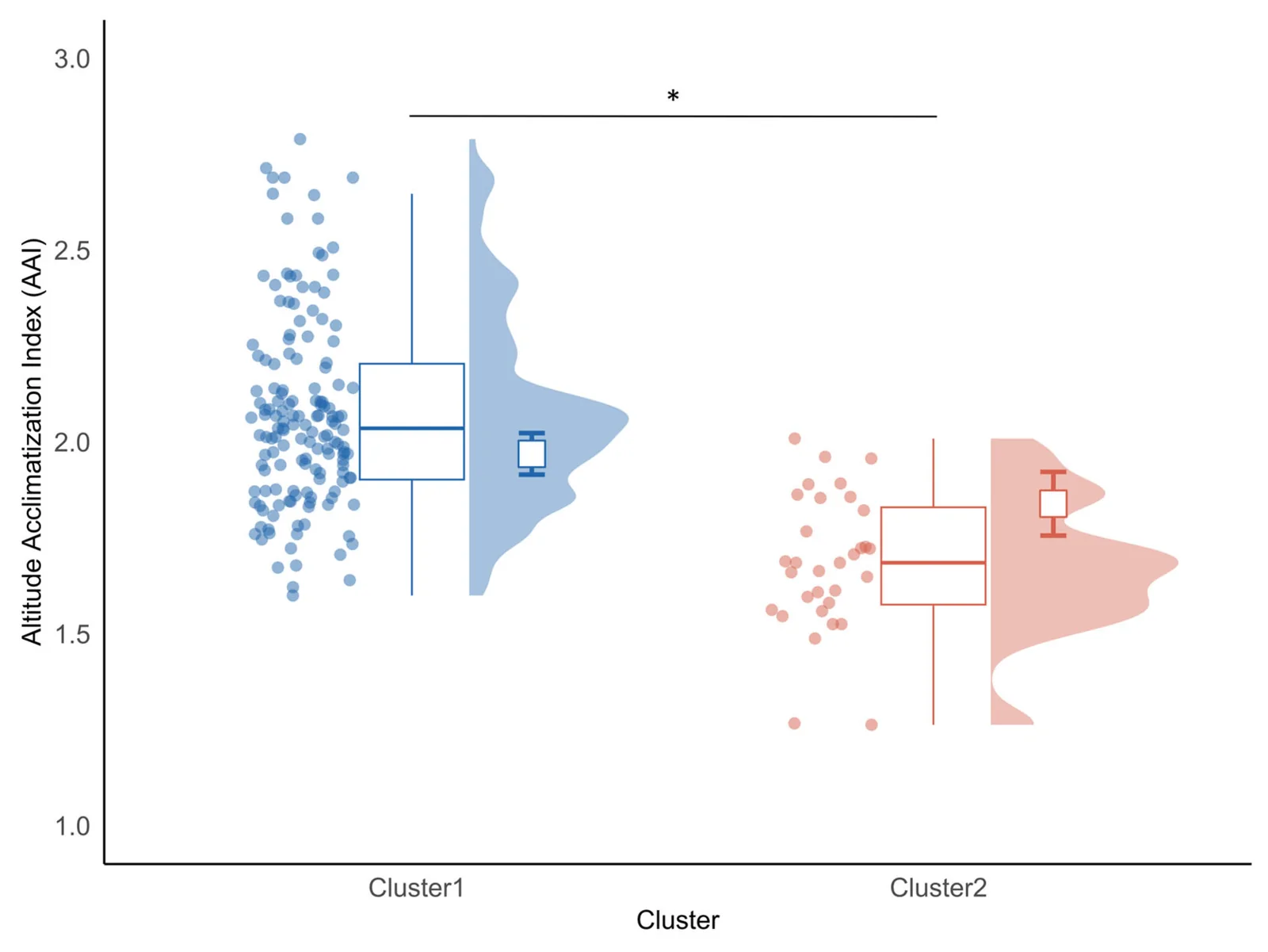
Raincloud plot of the AAI for the two clusters. Note: Combined jittered scatter, box, and half-violin plots show individual data, median with interquartile range, and density distribution. Small squares with error bars indicate estimated marginal means and 95% confidence intervals from an ANCOVA model adjusted for sex, race, age, and BMI. * *p* < 0.05 (Type III ANOVA with Tukey adjustment).

**Table 1:** Baseline characteristics of the two clusters.

Variable	Cluster 1 (*n* = 152)	Cluster 2 (*n* = 32)	*p*
Biochemical markers			
Urea (mmol/L)	4.66 ± 1.16	5.28 ± 1.37	0.030
Creatinine, CR (μmol/L)	61.47 ± 8.88	84.53 ± 10.13	<0.001
Uric acid, UA (μmol/L)	310.55 ± 79.05	463.75 ± 123.50	<0.001
ALT (U/L)	17.33 ± 7.21	47.74 ± 27.69	<0.001
AST (U/L)	19.55 ± 4.23	31.80 ± 12.00	<0.001
AST/ALT ratio	1.25 ± 0.38	0.80 ± 0.34	<0.001
Hemoglobin, Hb (g/L)	152.30 ± 17.80	185.75 ± 17.54	<0.001
Total protein, TP (g/L)	74.65 ± 3.51	75.49 ± 5.36	0.425
Albumin, ALB (g/L)	45.78 ± 2.31	47.60 ± 2.54	<0.001
Globulin, GLOB (g/L)	28.87 ± 2.90	27.89 ± 4.08	0.235
Total bilirubin, TBIL (μmol/L)	12.63 ± 6.01	21.09 ± 9.92	<0.001
Direct bilirubin, DBIL (μmol/L)	4.47 ± 2.14	7.21 ± 3.19	<0.001
Indirect bilirubin, IBIL (μmol/L)	8.15 ± 4.04	13.88 ± 7.10	<0.001
Altitude acclimatization parameters			
SpO_2_ (%)	91.41 ± 2.77	90.31 ± 2.76	0.059
HCT (%)	44.66 ± 4.67	54.13 ± 5.41	<0.001
AAI	1.96 ± 0.27	1.83 ± 0.21	0.038
Demographic characteristics			
Age (years)	29.45 ± 7.25	31.03 ± 12.76	0.502
BMI (kg/m^2^)	22.44 ± 3.64	26.18 ± 3.80	<0.001
Sex, male (%)	13 (8.6%)	30 (93.8%)	<0.001
Race, migrant (%)	72 (47.4%)	22 (68.8%)	0.044

Note: Data are presented as mean ± standard deviation (*M* ± SD) for continuous variables and as number (percentage) for categorical variables. Continuous variables were compared using Welch’s two-sample *t*-test. Non-normally distributed variables were additionally analyzed using Mann–Whitney *U* tests, with consistent results. Categorical variables were compared using Fisher’s exact test. *p* values were adjusted for multiple comparisons using the Benjamini–Hochberg false discovery rate (FDR) method.

## Data Availability

The data presented in this study are available on request from the corresponding author.

## Supplementary Materials

The following supporting information can be downloaded at https://www.mdpi.com/article/10.3390/life16101595/s1: Figure S1: Line plot of silhouette coefficients; Figure S2: Residual diagnostic plots for the ANCOVA model. (a) Residuals versus fitted values; (b) Normal Q-Q plot of residuals.

## Abbreviations

AAI	Altitude acclimatization/adaptation index
SpO_2_	Oxygen saturation
HCT	Hematocrit
CR	Creatinine
UA	Uric acid
ALT	Alanine aminotransferase
AST	Aspartate aminotransferase
TBIL	Total bilirubin
Hb	Hemoglobin
ARI	Adjusted rand index
PAM	Partitioning around medoids
PCA	Principal component analysis
FDR	False discovery rate
ANCOVA	Analysis of covariance
BMI	Body mass index
Q-Q	Quantile-quantile
DBIL	Direct bilirubin
IBIL	Indirect bilirubin
ALB	Albumin
TP	Total protein
GLOB	Globulin

## References

[1] Gatterer H., Villafuerte F.C., Ulrich S., Bhandari S.S., Keyes L.E., Burtscher M. (2024). Altitude illnesses. Nat. Rev. Dis. Primers.

[2] Zhang J., Guo S., Dou B., Liu Y., Wang X., Jiao Y., Li Q., Li Y., Chen H. (2025). High-Altitude Hypoxia Injury: Systemic Mechanisms and Intervention Strategies on Immune and Inflammatory Responses. Antioxidants.

[3] Zhao Q., Hao D., Wang S., Chen S., Zhou C., Fan C., Su Q., Huang W., Liu J., Kong Q. (2025). Exposure to high altitude leads to disturbances in host metabolic homeostasis: Study of the effects of hypoxia-reoxygenation and the associations between the microbiome and metabolome. mSystems.

[4] Kang P., Cheng F. (2026). Mechanisms and therapeutic prospects of hypoxia-inducible factor 1-alpha in acute kidney injury: A systematic review. Front. Cell Dev. Biol..

[5] Zhao Y., Wang Y., Li F., Yu G. (2026). Hypoxia-inducible factors in the immunometabolism of metabolic dysfunction-associated steatotic liver disease (MASLD): Molecular mechanisms and therapeutic implications. Front. Physiol..

[6] Soliman M.M., Aldhahrani A., Althobaiti F., Ahmed M.M., Sayed S., Alotaibi S., Shukry M., El-Shehawi A.M. (2022). Characterization of the Impacts of Living at High Altitude in Taif: Oxidative Stress Biomarker Alterations and Immunohistochemical Changes. Curr. Issues Mol. Biol..

[7] Ray S.C., Mason J., O’Connor P.M. (2019). Ischemic Renal Injury: Can Renal Anatomy and Associated Vascular Congestion Explain Why the Medulla and Not the Cortex Is Where the Trouble Starts?. Semin. Nephrol..

[8] Ibrahim E., Sohail S.K., Ihunwo A., Eid R.A., Al-Shahrani Y., Rezigalla A.A. (2025). Effect of high-altitude hypoxia on function and cytoarchitecture of rats’ liver. Sci. Rep..

[9] Pu L., Xu H., Wang Z., Li R., Ai C., Song X., Zhang L., Cheng X., Wang G., Wang X. (2024). Intermittent high altitude hypoxia induced liver and kidney injury leading to hyperuricemia. Arch. Biochem. Biophys..

[10] Zhang L., Feng E., Baima S., Laba C., Suolang L., Cang J., De Q. (2025). High altitude renal syndrome: Four elements or one source?. Ren. Fail..

[11] Liu C., Luo M., Benardini S., Ji X., Xiao Y., Luo Y., Yan W., Zheng F., Li Z.A., Niu C. (2026). Development of a high-altitude renal disease diagnostic model based on machine learning and multiple biomarker detection: A retrospective study of 19,068 patients. BMC Nephrol..

[12] Lee H.A., Seo Y.S. (2022). Current knowledge about biomarkers of acute kidney injury in liver cirrhosis. Clin. Mol. Hepatol..

[13] Yang H., Chen Y., He J., Li Y., Feng Y. (2025). Advances in the diagnosis of early biomarkers for acute kidney injury: A literature review. BMC Nephrol..

[14] Liang H., Song K. (2023). Comprehensive metabolomics and transcriptomics analysis reveals protein and amino acid metabolic characteristics in liver tissue under chronic hypoxia. PLoS ONE.

[15] Wang S.Y., Gao J., Zhao J.H. (2022). Effects of high altitude on renal physiology and kidney diseases. Front. Physiol..

[16] Goldfarb-Rumyantzev A.S., Alper S.L. (2014). Short-term responses of the kidney to high altitude in mountain climbers. Nephrol. Dial. Transplant..

[17] Palubiski L.M., O’Halloran K.D., O’Neill J. (2020). Renal Physiological Adaptation to High Altitude: A Systematic Review. Front. Physiol..

[18] Chen L., He X., Wang H., Hu S., Zhao H., Ni G., Yan H., Chen L., Deng C., Luo F. (2026). Physiological and molecular dynamic changes during 23-day high-altitude exposure reveal novel biomarkers for acclimatization. Front. Physiol..

[19] Talks B.J., Campbell C., Larcombe S.J., Marlow L., Finnegan S.L., Lewis C.T., Lucas S.J.E., Harrison O.K., Pattinson K.T.S. (2022). Baseline Psychological Traits Contribute to Lake Louise Acute Mountain Sickness Score at High Altitude. High Alt. Med. Biol..

[20] Roach R.C., Hackett P.H., Oelz O., Bärtsch P., Luks A.M., MacInnis M.J., Baillie J.K. (2018). Lake Louise AMS Score Consensus Committee. The 2018 Lake Louise Acute Mountain Sickness Score. High Alt. Med. Biol..

[21] Frühauf A., Burtscher M., Pocecco E., Faulhaber M., Kopp M. (2016). Subjective assessment of acute mountain sickness: Investigating the relationship between the Lake Louise Self-Report, a visual analogue scale and psychological well-being scales. SpringerPlus.

[22] Meier D., Collet T.H., Locatelli I., Cornuz J., Kayser B., Simel D.L., Sartori C. (2017). Does This Patient Have Acute Mountain Sickness? The Rational Clinical Examination Systematic Review. JAMA.

[23] Li H., Liu X., Wu Y., Wang L., Su R., Ma H. (2024). Evaluation model of acclimatization and adaptation at high altitude. Chin. Sci. Bull..

[24] Pilato S., Mrakic-Sposta S., Verratti V., Santangelo C., di Giacomo S., Moffa S., Fontana A., Pietrangelo T., Ciampini F., Bonan S. (2025). Urineprint of high-altitude: Insights from analyses of urinary biomarkers and bio-physical-chemical features of extracellular vesicles. Biophys. Chem..

[25] Aggarwal C.C., Hinneburg A., Keim D.A. (2001). On the surprising behavior of distance metrics in high dimensional space. Proceedings of the International Conference on Database Theory.

[26] Ward J.H. (1963). Hierarchical grouping to optimize an objective function. J. Am. Stat. Assoc..

[27] Kwo P.Y., Cohen S.M., Lim J.K. (2017). ACG Clinical Guideline: Evaluation of Abnormal Liver Chemistries. Am. J. Gastroenterol..

[28] FitzGerald J.D., Dalbeth N., Mikuls T., Brignardello-Petersen R., Guyatt G., Abeles A.M., Gelber A.C., Harrold L.R., Khanna D., King C. (2020). 2020 American College of Rheumatology Guideline for the Management of Gout. Arthritis Care Res..

[29] Rousseeuw P.J. (1987). Silhouettes: A graphical aid to the interpretation and validation of cluster analysis. J. Comput. Appl. Math..

[30] Hennig C. (2007). Cluster-wise assessment of cluster stability. Comput. Stat. Data Anal..

[31] Zhao L., Huang Y., Tan X. (2024). Preexisting Hyperuricemia Before High-Altitude Ascent is Associated with a Slower Recovery of Estimated Glomerular Filtration Rate Following Descent. High Alt. Med. Biol..

[32] Furian M., Tannheimer M., Burtscher M. (2022). Effects of Acute Exposure and Acclimatization to High-Altitude on Oxygen Saturation and Related Cardiorespiratory Fitness in Health and Disease. J. Clin. Med..

[33] Tannheimer M., Lechner R. (2019). The correct measurement of oxygen saturation at high altitude. Sleep Breath.

[34] Yang J., Jin Z.B., Chen J., Huang X.F., Li X.M., Liang Y.B., Mao J.Y., Chen X., Zheng Z., Bakshi A. (2017). Genetic signatures of high-altitude adaptation in Tibetans. Proc. Natl. Acad. Sci. USA.

[35] Murray A.J., Montgomery H.E., Feelisch M., Grocott M.P.W., Martin D.S. (2018). Metabolic adjustment to high-altitude hypoxia: From genetic signals to physiological implications. Biochem. Soc. Trans..

[36] O’Brien K.A., Simonson T.S., Murray A.J. (2020). Metabolic adaptation to high altitude. Curr. Opin. Endocr. Metab. Res..

[37] Pena E., El Alam S., Siques P., Brito J. (2022). Oxidative Stress and Diseases Associated with High-Altitude Exposure. Antioxidants.

[38] Yang J., Jia Z., Song X., Shi J., Wang X., Zhao X., He K. (2022). Proteomic and clinical biomarkers for acute mountain sickness in a longitudinal cohort. Commun. Biol..

[39] Yin R., Wu Y., Li M., Liu C., Pu X., Yi W. (2024). Association between high-altitude polycythemia and hypertension: A cross-sectional study in adults at Tibetan ultrahigh altitudes. J. Hum. Hypertens..

[40] Meng D.D., Kang Y.D., Chang D.H. (2025). Research on related factors of hyperuricemia in high altitude area migrant population. Front. Endocrinol..

[41] Lorenzo F.R., Huff C., Myllymäki M., Olenchock B., Swierczek S., Tashi T., Gordeuk V., Wuren T., Ri-Li G., McClain D.A. (2014). A genetic mechanism for Tibetan high-altitude adaptation. Nat. Genet..

[42] Chen X., Liu B., Deng Y., Yang F., Wang W., Lin X., Yu L., Pu H., Zhang P., Li Z. (2022). Cardiac Adaptation to Prolonged High Altitude Migration Assessed by Speckle Tracking Echocardiography. Front. Cardiovasc. Med..

[43] He Y., Zheng W., Guo Y., Yue T., Cui C., Ouzhuluobu, Zhang H., Liu K., Yang Z., Wu T. (2023). Deep phenotyping of 11,880 highlanders reveals novel adaptive traits in native Tibetans. iScience.

[44] Zhang X., Shi Q. (2026). Exposure to high altitude: A potential driver of accelerated biological aging. MedScience.

